# Development and assessment of a hospital admissions-based syndromic surveillance system for COVID-19 in Ontario, Canada: ACES Pandemic Tracker

**DOI:** 10.1186/s12889-021-11303-9

**Published:** 2021-06-26

**Authors:** Nicholas Papadomanolakis-Pakis, Allison Maier, Adam van Dijk, Nancy VanStone, Kieran Michael Moore

**Affiliations:** 1 Knowledge Management Division, Kingston, Frontenac and Lennox & Addington Public Health, 221 Portsmouth Avenue, Kingston, Ontario K7M 1V5 Canada; 2 Office of the Medical Officer of Health, Kingston, Frontenac and Lennox & Addington Public Health, 221 Portsmouth Avenue, Kingston, Ontario K7M 1V5 Canada

**Keywords:** Public health surveillance, Syndromic surveillance, Surveillance system, COVID-19, Hospitalizations, Hospital admissions, Situational awareness, Pandemic

## Abstract

**Background:**

The COVID-19 pandemic has continued to pose a major global public health risk. The importance of public health surveillance systems to monitor the spread and impact of COVID-19 has been well demonstrated. The purpose of this study was to describe the development and effectiveness of a real-time public health syndromic surveillance system (ACES Pandemic Tracker) as an early warning system and to provide situational awareness in response to the COVID-19 pandemic in Ontario, Canada.

**Methods:**

We used hospital admissions data from the Acute Care Enhanced Surveillance (ACES) system to collect data on pre-defined groupings of symptoms (syndromes of interest; SOI) that may be related to COVID-19 from 131 hospitals across Ontario. To evaluate which SOI for suspected COVID-19 admissions were best correlated with laboratory confirmed admissions, laboratory confirmed COVID-19 hospital admissions data were collected from the Ontario Ministry of Health. Correlations and time-series lag analysis between suspected and confirmed COVID-19 hospital admissions were calculated. Data used for analyses covered the period between March 1, 2020 and September 21, 2020.

**Results:**

Between March 1, 2020 and September 21, 2020, ACES Pandemic Tracker identified 22,075 suspected COVID-19 hospital admissions (150 per 100,000 population) in Ontario. After correlation analysis, we found laboratory-confirmed hospital admissions for COVID-19 were strongly and significantly correlated with suspected COVID-19 hospital admissions when SOI were included (Spearman’s rho = 0.617) and suspected COVID-19 admissions when SOI were excluded (Spearman’s rho = 0.867). Weak to moderate significant correlations were found among individual SOI. Laboratory confirmed COVID-19 hospital admissions lagged in reporting by 3 days compared with suspected COVID-19 admissions when SOI were excluded.

**Conclusions:**

Our results demonstrate the utility of a hospital admissions syndromic surveillance system to monitor and identify potential surges in severe COVID-19 infection within the community in a timely manner and provide situational awareness to inform preventive and preparatory health interventions.

**Supplementary Information:**

The online version contains supplementary material available at 10.1186/s12889-021-11303-9.

## Background

The COVID-19 pandemic continues to pose a major public health risk globally. As of May 2021, more than 157 million confirmed cases of COVID-19, including over 3.2 million (2.1%) deaths, have been reported to the World Health Organization since December 2019 [1]. In Ontario, Canada’s most populous province, there have been more than 486,200 confirmed COVID-19 cases, including over 23,500 (4.8%) hospitalizations and over 8200 (1.7%) deaths as of May 2021 [2].

The importance of public health surveillance systems to monitor the spread and impact of disease within the population has been well demonstrated during the COVID-19 pandemic [3–6]. Public health surveillance systems have the capability to serve as early warning systems and provide situational awareness during public health emergencies, including communicable disease outbreaks, natural disasters and bioterrorism, among others [7, 8]. Information from public health surveillance systems can also provide scientific evidence essential to public health decision-making and control measures. Additionally, public health surveillance can guide health-related policy development, including disease prevention and risk mitigation strategies, and contribute to epidemiologic understanding of various communicable and non-communicable diseases [7, 8].

Real-time syndromic surveillance (SyS) is one type of public health surveillance tool that uses pre-diagnostic health indicators discernable before diagnostic confirmation as an alert of changes in disease activity in the population [9]. For example, SyS systems can rely on signs, symptoms or preliminary diagnoses to monitor disease activity within the population and apply statistical methods to indicate a potential outbreak or other public health concern. Real-time SyS systems can facilitate rapid investigation of potential disease outbreaks or elevations in specific disease-related illnesses [8, 9]. Data sources for large scale regional SyS systems often include health or pseudo-health information from healthcare services such as emergency department (ED) [10, 11] or primary care visits [12–14], but could also include information from retail pharmaceutical sales [15, 16], emergency medical services dispatch data [17–21], telehealth phone lines [22, 23] and non-health information from social media [6, 24]. The indirect nature of health-related indicators, although nonspecific [25], have the ability to improve timeliness and sensitivity of data collection and provide flexibility to adapt to different illnesses and situations [7, 26].

The significance of real-time SyS surveillance has been demonstrated with previous threats to global health (i.e. bioterrorism, pandemics) such as SARS, H1N1 influenza and Ebola [22, 27–35]. Most recently, several SyS systems have been implemented globally to help monitor COVID-19 including the Centers for Disease Control and Prevention’s COVID Tracker [36] and a smartphone application to gather COVID-like illness data to identify potential clusters of COVID-19 in the United States [37], COOPERA (Covid-19: Operation for Personalized Empowerment to Render smart prevention And care seeking) which collects crowdsourced data in Japan [38, 39] and a participatory SyS tool for tracking COVID-19 using self-report syndromic data in Bangladesh [40], among others.

In response to the pandemic threat of SARS in 2003, Kingston, Frontenac and Lennox & Addington (KFL&A) Public Health developed the Acute Care Enhanced Surveillance (ACES) system in Ontario, Canada to improve the provincial public health system’s ability to monitor, prevent and respond to future communicable and non-communicable disease outbreaks [41]. ACES is a province-wide system that monitors hospital registration records for ED visits in real-time for more than 95% of Ontario’s acute care hospitals and nearly 80% of inpatient admissions records. ACES monitors 84 distinct syndromes; 24 are regularly validated against ICD-10 codes.

The objective of this study was to describe the development and effectiveness of a real-time public health SyS system – ACES Pandemic Tracker – as an early warning system and to provide situational awareness in response to the COVID-19 pandemic in Ontario, Canada using hospital admissions data.

## Methods

### Development of ACES pandemic tracker

#### Data sources

ACES Pandemic Tracker [42] uses hospital admissions data from the ACES system [41]. ACES collects patient data in real-time including date, time, age, sex, first 5 digits of postal code, reason for visit or admission and Canadian Triage Acuity Score; no direct personal identifiers are collected, such as name or health card number.

ACES applies natural language processing (NLP) to categorize words or phrases from the “reason for admission” or “chief complaint” into predefined syndromes. The use of chief-complaints, syndromes or symptoms collected as text have been cited as an advantage for public health surveillance since they are available more closely to real-time than diagnostic indicators such as ICD codes [43–45]. The NLP algorithms were developed by a team of content experts (acute care physicians and epidemiologists) that manually classified a large dataset of patient triage records into syndromes based on their chief complaint. The algorithms do not rely on keyword searches, but rather probabilistic decisions based on attaching learned weighting values to each word, part of a word, or phrase in the chief complaint. The performance of each syndrome was previously validated against diagnostic hospital records.

#### Syndromes of interest (SOI)

Syndromes are pre-defined groupings of symptoms or health indicators that may indicate a clinical diagnosis or health outcome. The following syndromes were initially used to detect possible cases of COVID-19 at time of hospital admission: asthma (AST), congestive heart failure (CHF), chronic obstructive pulmonary disorder (COPD), influenza-like illness (ILI), general infection (INF), pneumonia (PN), respiratory illness (RESP) and sepsis (SEP). These syndromes were chosen as they encompassed many of the symptoms described in patients that had contracted COVID-19 at the start of the pandemic. Aggregate numbers of admissions for all SOI and overall admission trends were examined to assess the specificity of syndromes and remove those experiencing significant decreases in hospital admissions related to the COVID-19 pandemic. After correlation analysis with laboratory confirmed cases, several aforementioned syndromes were removed as they were complicating the interpretation of COVID-19 surveillance data [46]. Thus, the most recent version of ACES Pandemic Tracker, as discussed in this paper, includes three SOI: pneumonia, influenza-like illness and general infection (see Additional file 1 for descriptions of symptoms captured in each SOI including ICD-10 codes used for validation).

Possible cases of COVID-19 were flagged as “suspect COVID-19 counts” identified by COVID-19 keywords (“covid*” OR “coronav*” OR “ncov”) and included “susp” or SOI. Suspected COVID-19 cases identified by both COVID-19 keywords and SOI are hereinafter referred to as suspected COVID-19 (including SOI), whereas suspected COVID-19 cases identified using only COVID-19 keywords are referred to as suspected COVID-19 (excluding SOI). Each patient was only counted once, even if they had more than one related syndrome (see Additional file 2 for full COVID-19 flagging criteria). All free-text admissions were reviewed bi-weekly to determine how COVID-related admissions were being captured in order to adapt to changing free-text patterns over time. Data presented in this manuscript are based on the final SOI inclusion criteria. The change in case numbers flagged due to bi-weekly review updates was minimal and likely to have a negligible impact on case numbers flagged in real-time as opposed to post-event analysis. The total number of admissions for all SOI were calculated daily.

#### Baseline data

We used 2 years of historical hospital admissions data (2018 and 2019) to compare recent trends of hospital admissions at the regional-level to what would be expected under normal conditions. Since there is no historical data for COVID-19 flagged admissions, these admissions were grouped with all admissions for SOI in 2020 and compared to 2018–2019 SOI. For each pandemic day, the average number of admissions for the same pre-pandemic day in 2018 and 2019 were used for comparison. In order to reduce the risk of increased baseline data counts due to chance, 7- and 30-day moving averages were used [47]. We calculated historical 7- and 30-day moving averages by adding together syndromes related to COVID-19 during the 2018–2019 period to minimize short-term fluctuations and remove day-of-the-week/month variation. To account for random variation in comparisons with historical data, we calculated + 1 and + 2 standard deviation(s) from the historical average. Hospitals without 2 years of complete baseline admissions data for 2018 and 2019 were excluded from ACES Pandemic Tracker.

SOI related to COVID-19 are monitored for abnormal numbers of patients in comparison to historical baselines. A signal for possible anomalous regional hospital activity occurs when the pandemic moving average of hospital admissions counts is greater than + 1 standard deviation above the historical average, but less than or equal to + 2 standard deviations (Level 2). Highly anomalous activity is indicated when the pandemic moving average is greater than + 2 standard deviations above the historical pre-pandemic average (Level 3).

### Evaluation of ACES pandemic tracker

#### Descriptive analyses

We performed descriptive analyses using data from ACES Pandemic Tracker to calculate the median, interquartile range and incidence rate of daily suspected COVID-19 hospital admissions. We compared these data to confirmed COVID-19 admissions using data available from the Ontario Ministry of Health [48]. The date recorded for a confirmed COVID-19 admission was the date of reporting to public health following a confirmed laboratory test.

We used Spearman’s rho to examine correlations between confirmed and suspected COVID-19 admissions in Ontario between March 1, 2020 and September 21, 2020. We also compared each SOI with confirmed COVID-19 admissions to determine which SOIs demonstrated more favorable correlations with confirmed COVID-19 admissions.

In an effort to detect the potential delay between SyS flags for suspected COVID-19 admissions and confirmed COVID-19 admissions in Ontario, a cross-correlation function (CCF) with differencing was calculated between daily suspected COVID-19 admissions and daily confirmed COVID-19 admissions. On March 19, 2020, the Ontario Ministry of Health disseminated a province-wide directive for the use of suspected COVID-19 flags to improve monitoring of ED visits and hospital admissions for symptoms related to COVID-19. Therefore, we used data between March 19, 2020 and May 31, 2020 (end of first wave/peak) to calculate the CCF. Statistical analyses were conducted using R (R Foundation for Statistical Computing, Vienna, Austria). All analyses were conducted with aggregate data in accordance with the ethical and legal limitations defined by the data sharing agreements between ACES, public health agencies and the participating hospitals in Ontario and is exempt from Research Ethics Board review.

## Results

### Key characteristics

**Fig. 1:**
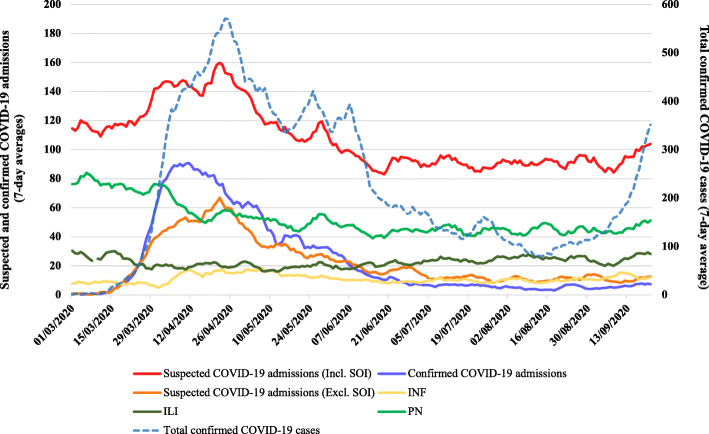
Trends in 7-day averages among suspected COVID-19 hospital admissions and confirmed COVID-19 cases between March 1, 2020 and September 21, 2020 in Ontario, Canada. *ILI* Influenza-like illness, *INF* General infection, *PN* Pneumonia, *SOI* Syndromes of interest. Note: Total confirmed cases (dotted-line) refers to the y-axis on the right

Between March 1, 2020 and September 21, 2020, ACES Pandemic Tracker identified 22,075 suspected COVID-19 hospital admissions, or 150 suspected COVID-19 admissions per 100,000 population among 131 hospitals in Ontario (Table 1). Of these suspected COVID-19 admissions, 48% were flagged as pneumonia, 21% as influenza-like illness, 20% as suspected COVID-19 (excluding SOI) and 10% as general infection. During the same time period, the province registered 47,268 confirmed cases of COVID-19, including 5047 COVID-19-related admissions, or 34 confirmed COVID-19 admissions per 100,000 population. A comparison of 7-day rolling averages of SOI admissions and confirmed COVID-19 cases is illustrated in Fig. 1.

**Table 1:** Counts for confirmed and suspected COVID-19 hospital admissions between March 1, 2020 and September 21, 2020

Syndrome	Total counts (%)	Median per day (IQR)
**Suspected COVID-19**		
Suspected COVID-19 admissions (including SOI)	22,075 (100%)	102 (91–124)
Suspected COVID-19 admissions (excluding SOI)	4457 (20%)	16 (10–30)
Pneumonia	10,690 (48%)	49 (43–57)
Influenza-like illness	4629 (21%)	22 (18–26)
General infection	2299 (10%)	11 (8–14)
**Confirmed COVID-19**		
Confirmed cases	47,268 (100%)	175 (111–370)
Confirmed admissions	5047 (11%)	9 (5–37)

Suspected COVID-19 is based on data from ACES Pandemic Tracker, whereas confirmed COVID-19 is based on data from the Ontario Ministry of Health

ACES Pandemic Tracker identified a median number of 102 (IQR 91–124) daily suspected COVID-19-related admissions (including SOI) in Ontario, including 49 daily suspected admissions for pneumonia (IQR 44–66), 22 for influenza-like illness (IQR 19–28), 16 for suspected COVID-19 (excluding SOI; IQR 10–31) and 11 for general infection (IQR 8–14; Table 1). The median number of confirmed COVID-19 admissions in Ontario during the same time period was 9 per day (IQR 5–37).

### Correlation analyses

We sought to determine whether suspected COVID-19 hospital admissions from ACES Pandemic Tracker were correlated with confirmed COVID-19 admissions from provincial data. We also calculated correlation coefficients for each SOI and suspected COVID-19 flag to determine which were more highly correlated with confirmed COVID-19 admissions.

Table 2 displays correlations between suspected and confirmed COVID-19 hospital admissions, including and excluding SOIs. We found suspected COVID-19 (including SOI) admissions to have a strong positive correlation with confirmed COVID-19 admissions (Spearman’s rho = 0.617). When SOI without COVID keywords were excluded, a stronger positive correlation was observed between suspected and confirmed COVID-19 admissions (Spearman’s rho = 0.867).

**Table 2:** Correlations between suspected COVID-19 hospital admissions/SOIs and confirmed COVID-19 admissions between March 1, 2020 and September 21, 2020

Syndrome	Spearman’s rho	***P*** value
Suspected COVID-19 admissions (including SOI)	0.617	<.0001
Suspected COVID-19 admissions (excluding SOI)	0.867	<.0001
General infection	0.311	<.0001
Influenza-like illness	−0.357	<.0001
Pneumonia	0.239	.0005

Spearman’s rho values ≥0.5 are considered to be highly correlated; 0.25 to 0.5 are considered to be moderately correlated and < 0.25 are considered to be weakly correlated

**Fig. 2:**
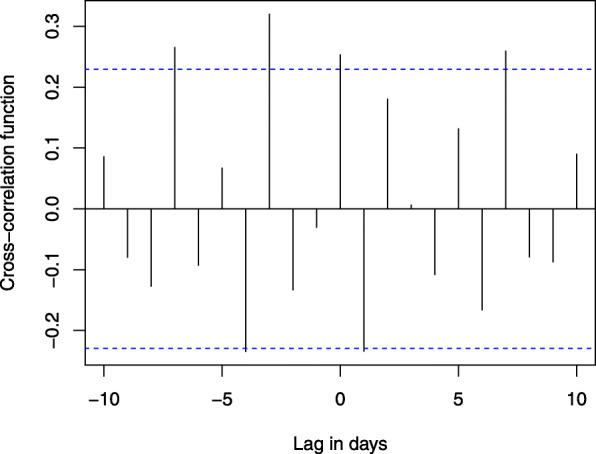
Time series lag analysis of suspected COVID-19 admissions compared with confirmed COVID-19 admissions in Ontario. The blue dotted lines represent 95% confidence intervals (CI) for lack of trend (0.229). Values that exceed these lines represent time-lagged trends. A CCF greater than the CI indicates a probable connection between the timing of suspected COVID-19 (excluding SOI) admissions and confirmed COVID-19 admissions. A lag of − 3 indicates that confirmed COVID-19 admissions lagged suspected COVID-19 admissions by 3 days. CCF values slightly greater than the CI at − 7 and 7 days are likely due to regular weekly fluctuations in hospital usage and acute/care patterns

Since suspected COVID-19 (excluding SOI) hospital admissions were most strongly correlated with confirmed COVID-19 admissions, we conducted a cross-correlation analysis to determine whether suspected COVID-19 (excluding SOI) admissions preceded confirmed COVID-19 admissions in Ontario, Canada. Our findings suggest confirmed COVID-19 admissions lagged suspected COVID-19 admissions by 3 days (CCF = 0.320). The time-series lag analysis is illustrated in Fig. 2.

## Discussion

This study described and evaluated the utility of ACES Pandemic Tracker, a province-wide real-time public health SyS tool. ACES Pandemic Tracker was developed to detect possible surges in severe COVID-19 infection requiring hospitalization and to provide situational awareness in Ontario, Canada. ACES Pandemic tracker uses hospital admissions data from 131 hospitals across the province to monitor syndromes related to COVID-19 with built-in spatial and temporal capabilities. Ontario has an estimated population of 14.7 million people; 39% of Canada’s population [49].

We found a strong correlation between suspected COVID-19 (including SOI) hospital admissions and confirmed COVID-19 admissions and a stronger correlation between suspected COVID-19 (excluding SOI) admissions and confirmed COVID-19 admissions. Although SOI can be valuable for identification of an unexpected disease outbreak, our findings suggest that suspected COVID-19 flags (excluding SOI) may be a more appropriate proxy for identifying COVID-19 infection when it is confirmed to be circulating within the population. These findings demonstrate that care should be taken when deciding whether to use pre-existing syndromes versus developing new flagging criteria for a novel disease outbreak. When SOI were assessed individually against confirmed COVID-19 admissions, we found a moderate positive correlation with suspected general infection admissions, a moderate negative correlation with suspected influenza-like illness admissions and a weak correlation with suspected pneumonia admissions. One possible explanation for the negative correlation with suspected influenza-like illness admissions is the overall decline in hospital patient volumes since COVID-19 restrictions were implemented in March 2020 [11, 50].

The negative effect of the COVID-19 pandemic on ED attendances has been demonstrated by Hughes et al. using Public Health England’s Emergency Department Syndromic Surveillance System (EDSSS) [11]. Compared to 2019 data, the authors determined ED attendances during March and April 2020 were significantly lower than ED attendances during the same time period in the previous year (non-respiratory indicators fell by 44–67% and acute respiratory infection fell by 4.4%) [11]. In relation to ACES Pandemic Tracker, syndromes often reported as suspected pneumonia and influenza-like illness admissions prior to the COVID-19 pandemic were instead being flagged by clinicians as suspected COVID-19 during the pandemic. This could be a result of recommendations circulated by the Ontario Ministry of Health to use suspected COVID-19 flags to improve surveillance and monitoring of COVID-19.

The cross-correlation analysis in the present study demonstrated a possible lag of 3 days between suspected COVID-19 (excluding SOI) admissions and confirmed COVID-19 admissions. Although this is likely related to the length of time required to report on laboratory results early in the pandemic, it illustrates the potential utility of using hospital admissions surveillance as an earlier indicator of COVID-19 severity when it is known to be circulating within the population. In terms of emergency preparedness, early warning of a potential surge in COVID-19 hospital admissions of 3 days can provide adequate time to re-allocate hospital resources including hospital staffing and availability of hospital beds in anticipation of severe COVID-19 infections that may require hospitalization and intensive care.

The SOI displayed in ACES Pandemic Tracker include symptoms that may be related to COVID-19 but are not clinical diagnoses. Correlation analyses between SOI and diagnosed COVID-19 cases need to be assessed on a regular basis and validated when diagnostic records are available, which are often offset by several days. The early warning capabilities built into ACES were exploited in the Pandemic Tracker based on aggregate counts of SOI and COVID-19 flagged hospital admissions to identify a potential surge in novel COVID-19 cases. SyS in this manner can provide situational awareness for epidemiologists, public health officials and hospitals to monitor for local analysis, improve identification and investigation and inform responses to possible surges in severe COVID-19 infection and other potential population health threats. It can also inform risk communication to the media, public and policy decision-makers.

Our results suggest one of the greatest strengths of ACES Pandemic Tracker is its ability to be easily updated with new keyword-based syndromes and adapted to new and emerging viruses and diseases which makes it a highly relevant and effective public health tool [33, 51]. ACES Pandemic Tracker monitors hospital admissions for syndromes related to COVID-19 since these presentations are expected to increase when widespread community infection of COVID-19 occurs. Improving the monitoring of syndromes related to COVID-19 admissions will assist in identification of potential cases and enable earlier physical distancing and self-isolation to limit the risk of person-to-person transmission and community spread of the virus. The incorporation of spatial information also allows public health professionals to identify specific regions that may have an increase in suspected COVID-19 infection rates.

Another strength of our surveillance approach lends itself to the monitoring of hospital admissions rather than ED visits. We believe surveillance of hospital admissions was more appropriate in this context since ED visits steadily declined from mid-March 2020. A possible explanation for the decline in ED visits in Ontario is that individuals who exhibited symptoms related to COVID-19 were directed to local assessment centers, which do not share data with ACES. Another explanation may simply be compliance with physical distancing measures. In addition, ED visit data was obscured by individuals who sought COVID-19 testing and other health-seeking behaviors during the initial wave of the COVID-19 pandemic which could lead to false assumptions regarding COVID-19 visits. Therefore, we believe hospital admissions data is a reliable source for surveillance of indications related to community spread of COVID-19 and resulting impacts on the healthcare system. Other international surveillance groups could consider adopting similar methods to monitor hospital admissions for future novel disease outbreaks. Furthermore, patients requiring hospital admission due to COVID-19 are generally more severe cases of infection that threaten to overwhelm hospital resources.

Overall, this study enumerates the statistical validity of ACES Pandemic Tracker and its usefulness as a disease surveillance system. The syndromic data provides a good estimation of real data counts that can inform public health action to reduce impact of a disease outbreak and community spread. Early in the pandemic, a limited number of public resources were available that could provide real-time information on COVID-19 cases and hospital admissions. The already established ACES system allowed for timely implementation of ACES Pandemic Tracker while more formalized provincial surveillance systems were under development. ACES Pandemic Tracker supported local public health agencies and other public health practitioners across the province as a standardized method to report hospital admissions data. The methodology, lessons learned and evidence from the post-hoc analyses demonstrate that a similar tool could be beneficial and adopted by other public health authorities in future pandemic scenarios.

Our findings are relevant for other national, regional and local public health agencies globally that may wish to establish and implement disease surveillance systems at a population level. Although our SyS was implemented in a Canadian setting, the methods used to monitor hospital admissions for possible increases in severe COVID-19 disease activity are not specific to one setting or disease. SyS of hospital admissions can be applied to other global contexts and to other novel disease outbreaks. Based on our results, we wish to emphasize that development of a new flagging criteria for a novel disease outbreak may be necessary under certain circumstances which may include a decline in baseline hospital volumes/syndromes or when disease presentation does not accurately reflect symptoms captured in existing syndromes. The new flagging criteria should be continuously updated to reflect possible changes or variation in disease presentation due to genetic drift over time. It should be noted that our approach required effective communication and collaboration among government, healthcare, public health agencies and the public. Globally, this may pose a barrier to implementation depending on institutional arrangements, cooperation, communication and public health resources [52].

There are limitations of ACES Pandemic Tracker that should be considered. First, we were unable to calculate sensitivity and specificity of the Pandemic Tracker since we did not have access to laboratory data. However, based on previous evaluations of syndromic surveillance systems, due to the non-differential nature of selected key syndromes and since laboratory confirmation is not required for suspected COVID-19 flags, we would expect Pandemic Tracker to be highly sensitive with low specificity, when compared to confirmed COVID-19 admissions [25]. Second, the value of ACES Pandemic Tracker was perhaps best demonstrated as an early warning system to help guide system planners while laboratory testing for COVID-19 was delayed and there were significant restrictions on testing prior to establishment of COVID-19 assessment centres. However, it maintains the ability to estimate the potential burden of severe COVID-19 cases on hospital resources.

## Conclusion

The ability to predict potential surges of severe COVID-19 and other communicable diseases within the population is of particular importance for public health surveillance, intervention and prevention. The evaluation of ACES Pandemic Tracker suggests SyS of hospital admissions could be used to provide situational awareness, monitor real-time patient volumes and identify potential surges in severe COVID-19 infection levels in a timely manner. Such SyS systems can be utilized by public health agencies, healthcare professionals, community service partners and testing laboratories to take informed measures to plan and allocate resources appropriately and aid public health in implementing measures to prevent further spread of infection.

## Supplementary Information

**Additional file 1.** Syndrome descriptions and ICD-10 codes for validation.

**Additional file 2.** COVID-19 flagging criteria.

## Data Availability

ACES is bound by data sharing agreements with each participating hospital and/or hospital corporation and their regional local public health agencies. ACES Pandemic Tracker is a publicly accessible tool and available without a user agreement enabling free and open information for academics, health professionals, emergency services, the media and the public on the community spread of COVID-19 and its impact on hospitals at https://www.kflaphi.ca/aces-pandemic-tracker/. Data for confirmed COVID-19 cases is publicly available and openly accessible from the Ontario Ministry of Health at https://covid-19.ontario.ca/data.

## Abbreviations

ACES: Acute Care Enhanced Surveillance
AST: Asthma
CCF: Cross-correlation function
CHF: Congestive heart failure
CI: Confidence interval
COPD: Chronic obstructive pulmonary disorder
COVID-19: Coronavirus disease 2019
ED: Emergency department
ICD: International classification of diseases
ILI: Influenza-like illness
KFL&A: Kingston, Frontenac and Lennox & Addington
NLP: Natural language processing
PN: Pneumonia
RESP: Respiratory
SARS: Severe acute respiratory syndrome
SEP: Sepsis
SOI: Syndrome of interest
SyS: Syndromic surveillance
