# Supplementary material for: Development and assessment of a hospital admissions-based syndromic surveillance system for COVID-19 in Ontario, Canada: ACES Pandemic Tracker
Source: BMC Public Health. 2021 Jun 26;21:1230. doi: 10.1186/s12889-021-11303-9 (PMC8233625; doi:10.1186/s12889-021-11303-9)
Supplement: Supplementary file 1 — Additional file 1. Syndrome descriptions and ICD-10 codes for validation. [file 12889_2021_11303_MOESM1_ESM.docx]

Syndrome descriptions and ICD-10 codes for validation.

| **ACES Code** | **Syndrome description** | **ICD-10 codes used for validation with Intellihealth (ED) or DAD (AD) patient records** |
| --- | --- | --- |
| **ILI** | Fever, myalgia, undifferentiated flu | R50, J10, J11 |
| **INF** | Non-specific infections: potential interest to public health, epiglottis, tonsil abscess | Not validated beyond algorithm framing |
| **PN** | Pneumonia | J12, J15, J16, J17, J18 |

*Bold indicates syndromes included in most recent version of Pandemic Tracker.

*AD* Admissions, *DAD* Discharge Abstract Database, *ED* Emergency department, *ILI* Influenza-like illness, *INF* General infection, *PN* Pneumonia.
