# Supplementary material for: Development and assessment of a hospital admissions-based syndromic surveillance system for COVID-19 in Ontario, Canada: ACES Pandemic Tracker
Source: BMC Public Health. 2021 Jun 26;21:1230. doi: 10.1186/s12889-021-11303-9 (PMC8233625; doi:10.1186/s12889-021-11303-9)
Supplement: Supplementary file 2 — Additional file 2. COVID-19 flagging criteria. [file 12889_2021_11303_MOESM2_ESM.docx]

COVID-19 flagging criteria

Admissions are flagged as COVID based on several criteria that are regularly reviewed and updated. The criteria are developed by reviewing the words in patient admissions records that are used by healthcare staff to identify COVID-19.

Currently, the criteria for an admission to be flagged as COVID include the following:

1. Identify all admissions that include the words/part of words “covid”, “coronav” (for coronavirus), and “ncov” in the reason for admission.
2. Remove any admissions that include keywords suggesting COVID is not probable (e.g., low risk or unlikely).
3. Keep only admissions that meet at least one of these additional criteria:
4. The word “susp” is included. Hospitals have been asked to identify potential COVID patients using the phrase “suspect covid”.
5. The admission is classified into a syndrome that often captures potential COVID cases. These syndromes include the syndromes used as baseline (e.g., pneumonia, asthma, influenza-like illness, and sepsis) and a few others (e.g., bronchitis and coronary artery disease).
6. The admission is classified into a generic syndrome (e.g., other or general surgery) and does not include keywords that suggest the patient was only being tested or screened for COVID (e.g., “query” or “rule out”).
7. The admission is classified into the infection syndrome, but does not include the keyword “unspecified” (this may refer to other coronaviruses).

Note: **Just because an admission has been flagged as COVID, it does not mean it represents a COVID-19 positive patient.** That cannot be known until the patient is tested and the laboratory result is returned. The COVID flag works to capture the most likely COVID-19 admissions, but will also capture patients with other illnesses.
